# Coronary microvascular dysfunction: pathophysiology, diagnosis, and therapeutic strategies across cardiovascular diseases

**DOI:** 10.17179/excli2025-8285

**Published:** 2025-03-26

**Authors:** Vincenzo Scarica, Riccardo Rinaldi, Francesco Maria Animati, Matteo Manzato, Rocco A. Montone

**Affiliations:** 1 Department of Cardiovascular and Pulmonary Sciences, Catholic University of the Sacred Heart, Rome, Italy; 2 Cardiology Unit, Infermi Hospital, Rimini, Italy; 3 Department of Cardiovascular Sciences, Fondazione Policlinico Universitario A. Gemelli IRCCS, Rome, Italy

**Keywords:** coronary microvascular dysfunction, INOCA, ANOCA, coronary spasm, precision medicine

## Abstract

Ischemic heart disease (IHD) is a leading cause of morbidity and mortality worldwide, presenting with acute and chronic coronary syndromes. Although coronary atherosclerosis is a major cause of IHD, many patients with angina or myocardial ischemia do not have obstructive coronary heart disease and impairment of the coronary microcirculation has been increasingly implicated as a relevant cause of IHD. Therefore, coronary microvascular dysfunction (CMD) refers to a term covering a wide spectrum of structural and functional alterations which affect the coronary microcirculation leading to myocardial ischemia and angina. The advent of non-invasive and invasive functional tests has exponentially broadened the ability to recognize CMD and delineate related clinical and biochemical features. Despite major advances in diagnosing and stratifying this condition, therapeutic strategies remain limited and poorly defined. In this review, we will provide an overview of the pathophysiology and the diagnostic evaluation of CMD across the spectrum of cardiovascular diseases. Furthermore, we will discuss the novel therapeutic strategies available for these patients in the perspective of a personalized medicine approach.

## Introduction

Myocardial infarction and myocardial ischemia have been traditionally considered a “large vessel” disease caused by atherosclerosis and obstructive atherothrombotic events in the epicardial coronary arteries. However, up to half of the subjects undergoing invasive coronary angiography for angina or non-invasive evidence of myocardial ischemia have non-obstructive coronary artery disease (Vrints et al., 2024[115]). In these patients impairment of coronary microvascular flow is an established cause of myocardial ischemia. Coronary microvascular dysfunction (CMD) encompasses several pathogenetic mechanisms resulting in functional and structural changes in the coronary microcirculation. CMD often determines angina and myocardial ischemia not only in patients with ischemia with non-obstructive coronary arteries (INOCA) or infarction with non-obstructive coronary arteries (MINOCA) but also in a broad spectrum of cardiovascular diseases, such as ischemia with obstructive coronary artery disease, cardiomyopathies, Takotsubo syndrome and heart failure, especially heart failure with preserved ejection fraction. 

The aim of this review is to provide updated evidence and a comprehensive overview of the complex pathophysiology of CMD, the available diagnostic techniques and the prognostic role of CMD across cardiovascular diseases. Moreover, it will include a detailed discussion about current clinical management of CMD with a focus on novel potential therapies and ongoing trials.

## Pathophysiology of CMD

The coronary arterial system consists of epicardial coronary vessels and coronary microcirculation. 

Epicardial coronary vessels are conductance arteries with a cross-sectional diameter >500 *μ*m, visible on coronary angiography and the site of obstructive atherosclerosis (Camici and Crea, 2007[10]).

**Figure 1:**
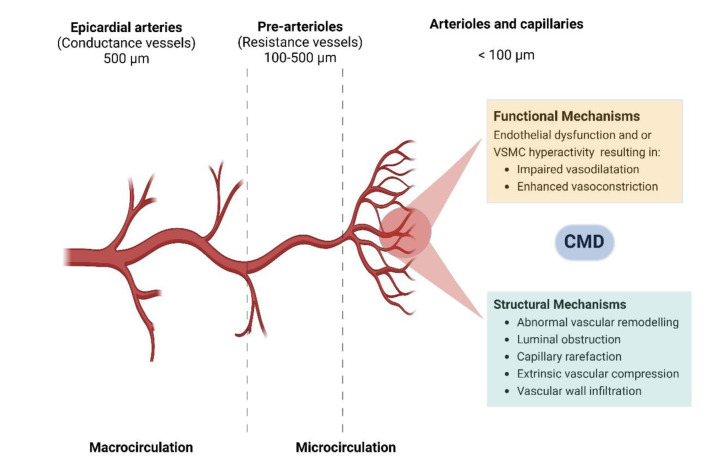
Pathophysiology of coronary microvascular dysfunction (*Abbreviations: CMD: coronary microvascular dysfunction; VSMC: vascular smooth muscle cells)*

Coronary microcirculation includes all vessels with a cross-sectional diameter <500 *μ*m such as the pre-arteriolar vessels (500-100 *μ*m diameter), intramural arterioles (diameter <100 *μ*m) and capillaries, which are resistive arteries being responsible for >70 % of the coronary resistance under physiological conditions (Crea et al., 2022[17]). In healthy individuals, without significant epicardial atherosclerosis, increases in myocardial metabolic demand are met by progressive vasodilation of coronary arterioles, which can normally induce a five-fold increase of coronary blood flow (Padro et al., 2020[79]). The mechanisms underlying CMD involve the combination of both structural, functional and molecular alterations in the coronary microcirculation, impairing the ability of the coronary microcirculation to increase coronary blood flow (CBF) in response to an increased myocardial oxygen demand or resulting in coronary microvascular spasm (Figure 1(Fig. 1)).

The structural changes of the coronary microcirculation responsible for CMD have been documented especially in patients with traditional cardiovascular risk factors for coronary heart disease (CAD) and different cardiomyopathies (Taqueti and Di Carli, 2018[107]). These alterations may include capillary rarefaction and adverse remodeling of intramural coronary arterioles, resulting from medial wall thickening mainly because of smooth muscle hypertrophy and increased collagen deposition causing perivascular fibrosis, with variable degrees of intimal thickening and a reduced wall/lumen ratio (Crea et al., 2022[17]). Clinical studies provided evidence that microvascular hallmarks of hypertension are inward remodeling of resistance arteries and microvascular rarefaction (Triantafyllou et al., 2016[110]). In patients with hypertrophic cardiomyopathy (HCM), CMD mechanisms also include myocyte disarray and elevated left ventricular end-diastolic pressure (Cecchi et al., 2003[12]). Diabetes mellitus is also associated with myocardial vasoconstriction and reduced angiogenesis in both animals and humans (Hinkel et al., 2017[42]). These structural changes can, even in the absence of epicardial CAD, induce progressive reductions in coronary flow reserve (CFR) mimicking the effects of flow limiting stenosis. However, the difference is that CFR reduction in patients with CMD rarely follows the regional (in the territory subtended by the stenotic artery) pattern seen in patients with obstructive CAD, rather appearing as patchy, with small areas of ischemic tissue interspersed among otherwise normal myocardium, or diffuse, involving most of the ventricle (Kaski et al., 2018[46]; Crea and Montone, 2023[16]). 

The main functional abnormalities leading to CMD include impaired dilatation or excessive coronary microvascular constriction as well as autonomic dysfunction. Impaired vasodilatation may be because of abnormalities in endothelium-dependent mechanisms (frequently associated with diabetes mellitus, obesity, smoking, and other cardiovascular risk factors), endothelial-independent mechanisms, or both (Kaski et al., 2018[46]).

The endothelium, a mono-layer of endothelial cells, modulates vascular tone by synthesizing and liberating endothelium-derived relaxing factors (EDRFs), including vasodilator prostaglandins (e.g. prostacyclin), nitric oxide (NO), and endothelium-dependent hyperpolarization (EDH) factors as well as endothelium-derived contracting factors (EDCFs) in response to shear stress and various agonists *in vivo*. EDH-mediated responses are accompanied with hyperpolarization and relaxation of the underlying vascular smooth muscle cells (VSMC) and subsequent vasodilatation (Godo et al., 2023[35]). It is widely accepted that EDH factors, rather than NO, predominantly mediate the endothelium-dependent vasodilatation of resistance arteries (Godo et al., 2021[36]; Vanhoutte et al., 2017[113]). Endothelium-independent mechanisms involve increased release of vasoconstrictor agonists, such as Endothelin-1 (ET-1), increased susceptibility of VSMCs to normal vasoconstrictor stimuli, decreased VSMC relaxation and aberrant autonomic activity (Crea et al., 2022[17]). Autonomic dysfunction involves increased sympathetic activation. However, increased alpha-adrenergic coronary vasoconstriction is clinically relevant when non-neural vasodilator mechanisms are impaired (such as patients with metabolic syndrome, dyslipidemia or type 2 diabetes mellitus, and in the acute phase after myocardial infarction or percutaneous revascularization procedures) (Crea et al., 2022[17]; Grassi et al., 2007[38]; Schelbert, 2010[99]). Among functional alterations, more recent evidence supports the hypothesis that air pollution could contribute to coronary microvascular disorder. Exposure to particulate matter (PM2.5 and PM10) is independently associated with coronary vasomotor disorders. More precisely, PM2.5 and PM10 are associated with a positive coronary provocation test, although epicardial spasms seem to occur more frequently than microvascular spasms (Camilli et al., 2022[11]).

**Figure 2:**
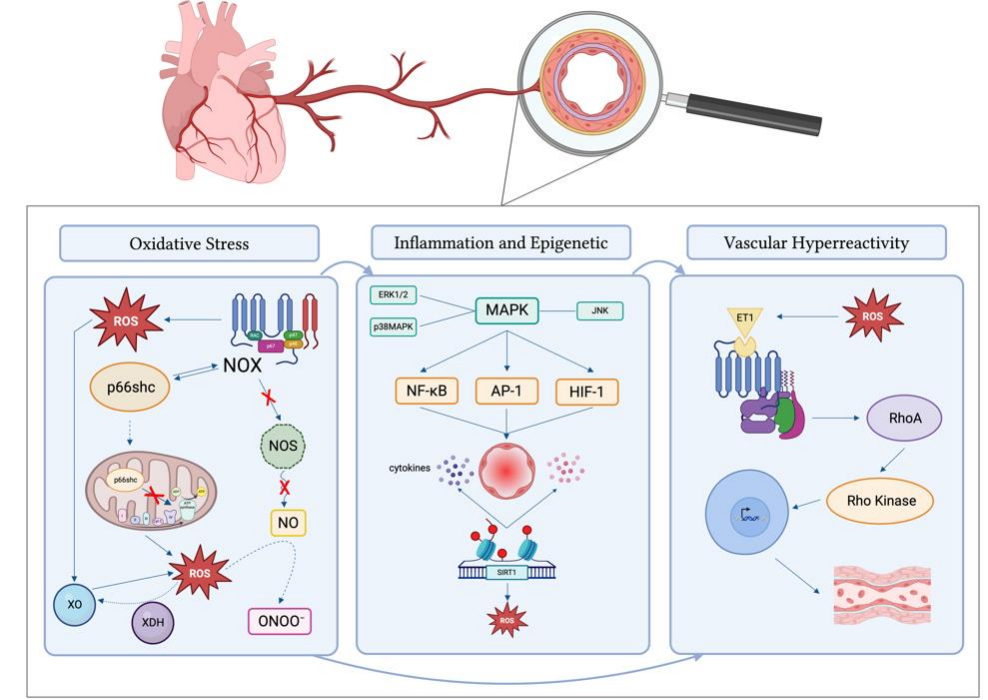
Molecular pathways involved in coronary microvascular dysfunction Abbreviations. AP-1: Activator Protein 1; ERK1/2: Extracellular Signal-Regulated Kinases 1 and 2; ET1: Endothelin 1; HIF-1: Hypoxia-Inducible Factor 1; JNK: c-Jun N-terminal Kinase; MAPK: Mitogen-Activated Protein Kinase; NF-κB: Nuclear Factor Kappa B; NO: Nitric Oxide; NOS: Nitric Oxide Synthase; NOX: NADPH Oxidase; ONOO⁻: Peroxynitrite; p38MAPK: p38 Mitogen-Activated Protein Kinase; p66shc: SHC Adaptor Protein 1; Rho Kinase: Rho-Associated Protein Kinase; RhoA: Ras Homolog Family Member A; ROS: Reactive Oxygen Species; SIRT1: Sirtuin 1; XO: Xanthine Oxidase; XDH: Xanthine Dehydrogenase.

**Figure 3:**
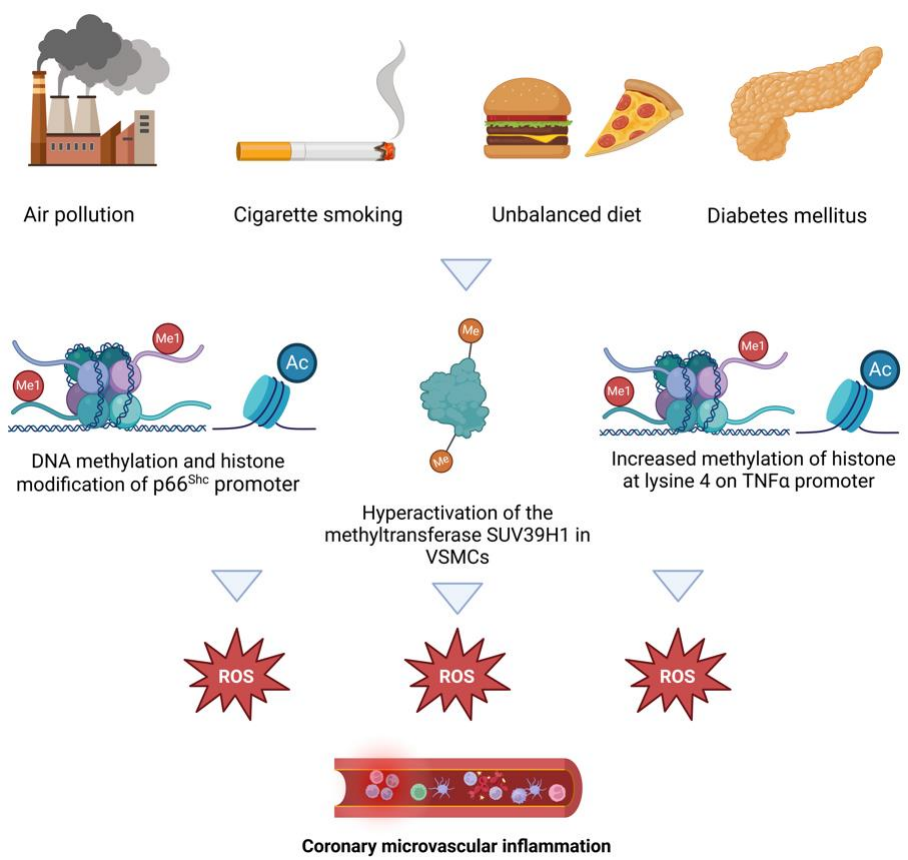
Epigenetic modifications involved in coronary microvascular dysfunction Abbreviations. TNFα: Tumor necrosis factor; VSMC: vascular smooth muscle cells

Molecular pathways associated with CMD include increased oxidative stress, impaired RhoA/Rho-kinase activity and epigenetic modifications (Crea and Montone, 2023[16]). Reactive oxygen species (ROS) and inflammatory responses play a central role in the pathogenesis of CMD. ROS production is predominantly regulated by nicotinamide adenine dinucleotide phosphate oxidases (NOx) family, nitric oxide synthase (NOS) uncoupling, xanthine oxidase and mitochondria (Zhang et al., 2023[120]). The activation of Nox enzymes leads to ROS production and triggers p66Shc phosphorylation and translocation within the mitochondria, where it further promotes ROS generation by altering the function of the mitochondria respiratory chain. In turn, p66Shc activation stimulates the activity of Nox, thus generating a vicious cycle that amplifies ROS production (Masi et al., 2021[55]). Moreover, ROS can inactivate NO to form a powerful oxidant, peroxynitrite, that can nitrose substrate proteins and uncouple eNOS to produce ROS; reactive oxygen species can also promote the conversion of xanthine dehydrogenase (XDH) to XO by oxidizing sulfhydryl residues, thereby inducing ROS production (Lassègue and Griendling, 2010[50]). ROS mediate microvascular aging, vasodilation dysfunction, and inflammatory response activating mitogen-activated protein kinases (ERK1/2, p38MAPK, and JNK), tyrosine kinases, and tyrosine phosphatases. Important downstream redox targets are transcription factors (NF-kB, AP-1 and HIF-1) and pro-inflammatory genes, critically involved in chemokine and cytokine production, recruitment, and activation of inflammatory and immune cells, which promote microvascular inflammation (Masi et al., 2021[55]). ROS also enhance the coronary microvascular contractile activity of ET-1 by activating the RhoA/Rho kinase pathway (Lassègue and Griendling, 2010[50]) (Figure 2(Fig. 2)). Epigenetic modifications contribute to the oxidative stress-mediated CMD; DNA methylation and histone modifications of the p66^Shc ^gene and the TNF α promoter are the most studied epigenetic alteration involved in obesity, diabetes and hypertension-related CMD. Likewise, endothelial inflammation is favored by mono-methylation of histone 3 and activation of the methyltransferase SUV39H1 via up-regulation of NF-kB. Commonly detected in ageing, reduced SIRT1 expression sustains a further increase in intracellular oxidative stress, reducing the expression of antioxidant enzymes (secondary to FOXO inhibition) and promoting pro-inflammatory cytokine (Masi et al., 2021[55]) (Figure 3(Fig. 3)).

## Diagnostic Techniques

Coronary microvasculature can be evaluated through both invasive and non-invasive techniques (Montone et al., 2021[64]). Table 1(Tab. 1) summarizes non-invasive techniques, whereas a more extensive discussion on invasive techniques is warranted in the following section.

### Invasive techniques

Coronary angiography allows to exclude significant obstructive stenosis and, by performing a comprehensive invasive functional assessment, diagnose the presence of functional and structural alteration of the microcirculation.

An initial visual assessment of CMD may be performed through Thrombolysis In Myocardial Infarction (TIMI) frame count, with delayed opacification (>25 frames), the so-called “coronary slow-flow phenomenon”, allowing to suspect the presence of CMD (Xu et al., 2022[118]).

The two cornerstone measurements in the assessment of endothelium-independent CMD are coronary flow reserve (CFR) and index of microcirculatory resistance (IMR).

CFR is defined as the ratio between adenosine-induced hyperemic blood flow to resting blood flow. This ratio quantifies the response to an increased oxygen demand. Adenosine is the preferred agent as the effect on epicardial vessels is minimal, thus affecting mainly the microcirculation. An effective alternative to Adenosine - in patients with contraindications to this molecule - is represented by Papaverine, although it is linked with a higher risk of polymorphic ventricular tachycardia (Nakayama et al., 2015[70]). Coronary flow may be assessed either by doppler, accounting for the change in velocity before and during hyperemia, or by thermodilution, using instead the mean transit time.

CFR is non-specific for the microcirculation, thus for a correct interpretation is essential to exclude the presence of obstructive epicardial CAD. A cut-off value of 2.0 has been conventionally accepted for the diagnosis of CMD (Ong et al., 2018[78]), although Doppler-obtained CFR may have a less stringent cut-off of 2.5.

IMR is a microcirculation-specific parameter, as the majority of coronary arteries resistance originates in the arteriolar system. This parameter is obtained by thermodilution, calculated as the product of pressure distal to the catheter and mean transit time of cold saline flush at hyperemia (Fearon et al., 2003[28]). Values > 25 are indicative for a diagnosis of CMD.

Hyperemic microvascular resistance (hMR) uses a combined pressure and Doppler flow catheter, measuring the mean distal pressure to average peak blood flow velocity ratio (Meuwissen et al., 2001[57]). hMR showed moderate correlation with IMR (Williams et al., 2018[117]), and a value greater than 2.5 mmHg/ cm/s is predictive of CMD (Feenstra et al., 2023[29]).

Based on the results of invasive coronary functional tests, some studies propose a division of CMD patients in two categories: a first group with “structural or functional CMD”, which is characterized by negative Ach test and impaired CFR, IMR or both and a second group with microvascular spasm.

This group can be further divided into two other categories:

A “structural” CMD, defined by high IMR and reduced CFR indexes.

A “functional” CMD, defined by normal IMR and reduced CFR indexes.

Structural CMD endotypes are more often linked to a higher likelihood of acute coronary syndromes and higher mortality. In contrast, functional CMD is more commonly associated with an increased risk of hospitalizations secondary to recurrent episodes of angina (Rahman et al., 2020[87]).

The second group is constituted by individuals with evidence of microvascular spasm - id est arteriolar dynamic obstruction - and is diagnosed by a positive Ach test associated with epicardial vessel diameter reduction <90 % (Montone et al., 2024[67]).

Both endotypes of CMD can coexist in patients with microvascular angina (MVA). Their differentiation is crucial in order to offer the best tailored therapy to subjects with CMD (Montone et al., 2024[67]).

An assessment of endothelium-dependent CMD vasospasm through Acetylcholine (Ach) provocation testing is fundamental to exclude microvascular spasm. This is a proven safe procedure (Montone et al., 2022[66]) in which escalating doses of Ach - up to a maximum of 200 mcg - are injected into the left anterior descending artery (LAD). LAD is commonly considered the preferred injection site given its dominance on total coronary circulation and the significant extent of myocardial tissue supplied by this vessel. In addition to this, considering the not so uncommon risk of Ach induced bradycardia, right coronary artery or dominant circumflex artery injection are unsafe procedures and should be avoided (Byrne et al., 2023[8]). According with COVADIS criteria, epicardial spasm is defined by the occurrence of chest pain, ischemic ECG changes (i.e. new horizontal or downsloping ST-segment depression of ≥0.5 mm in at least two adjacent leads and/or T-wave inversion greater than 1 mm in two contiguous leads or new ST-segment elevation at the J-point ≥1 mm in at least two adjacent leads in all leads except V2-V3, where the criteria are ≥2 mm for men aged 40 or older, ≥2.5 mm for men younger than 40, and ≥1.5 mm for women of any age (Thygesen et al., 2018[108]) alongside an angiographic diameter reduction >90 % (Ong et al., 2018[78]). Conversely, microvascular spasm is diagnosed when chest pain and ECG changes occur with an angiographic diameter reduction <90 % (Vrints et al., 2024[115]). Microvascular spasm is associated with a better prognosis compared to macrovascular one (Rinaldi et al., 2022[93]). A recent study by Rinaldi et al. demonstrated that the incidence of major adverse cardiac and cerebrovascular events (MACCEs) in patients satisfying one or more COVADIS criteria is notably higher than in those without any positive criterion, mainly for rehospitalizations for unstable angina. According to the same study, MACCE-free survival is inversely proportional to overall COVADIS criteria number, with the best clinical outcomes observed in patients without positive criteria and the worst in those meeting all three (Rinaldi et al., 2025[92]).

Current ESC guidelines on chronic coronary syndromes recommend the use of invasive functional testing to extrapolate CFR and IMR - in patients with persistent chest pain despite medical treatment and normal coronary anatomy - in order to identify the correct endotype of CMD and offer a potential targeted therapy (CR: I, LOE: B) (Vrints et al., 2024[115]).

## Role of CMD Across Cardio-Vascular Diseases (Figure 4)

### CMD in patients with obstructive CAD 

Some patients with acute coronary syndrome (ACS) and obstructive CAD have an increased risk of major acute cardiovascular events (MACE) because of plaque morphology (rupture of the fibrous cap versus intact fibrous cap) (Gerhardt et al., 2023[34]) but also the coexistence of CMD with obstructive CAD has prognostic implications, both in chronic in acute settings. Indeed, a study from Van de Hoef et al. demonstrated that, in patients with obstructive CAD on diagnostic coronary angiography, a value of CFR assessed invasively ≤2.7 was associated with a 2.24-fold increase in all-cause mortality hazard (van de Hoef et al., 2013[111]). Similar findings were reported by non-invasive studies. Ankur Gupta et al. showed that in a population of 4029 patients, elevated cardiovascular mortality was independently driven by the impairment of CFR obtained through PET-scan (Gupta et al., 2017[39]).

Furthermore, CMD could be a significantly contributor to myocardial ischemia in patients with obstructive coronary artery disease (CAD) with persistent or recurrent angina following successful percutaneous coronary intervention (PCI) and coronary stenting. While PCI can offer substantial symptoms relief and improved quality of life (QoL) compared with optimal medical therapy (OMT) alone, angina persistence or recurrence after PCI remains a major challenge (Niccoli et al., 2017[73]). The ORBITA and ORBITA-2 trials showed that a significant proportion of patients (61 % in ORBITA and 59 % in ORBITA-2) continued to experience angina despite effective revascularization and OMT (Al-Lamee et al., 2018[3]; Nowbar et al., 2022[74]). Similarly, previous studies have reported a 20 % to 30 % likelihood of angina recurrence within the first year after PCI, increasing to approximately 40 % within 3 years (Ben-Yehuda et al., 2016[5]). Notably, Li et al. and Milo et al. found that a reduced CFR after successful PCI was associated with recurrent angina and abnormal functional stress testing (Li et al., 2015[51]; Milo et al., 2013[58]).

Another clinical scenario where CMD plays a pivotal role in obstructive CAD is coronary microvascular obstruction (CMVO). In the primary PCI setting, CMVO is the phenomenon where despite restoring patency of the epicardial coronary vessels in acute coronary syndrome, structural and functional abnormalities of the microvasculature result in suboptimal myocardial reperfusion (Rehan et al., 2023[90]). Experimental and clinical studies have shown that CMVO is caused by a combination of several pathogenic components including ischemic injury, reperfusion injury, distal atherothrombotic embolization, along with myocardial edema and/or inflammation leading to microvascular compression (Galli et al., 2024[33]). Bulluck et al. showed that patients with an IMR > 41 after primary PCI were more likely to have MVO at CMR, providing further evidence that CMVO is caused by CMD (Bulluck et al., 2016[7]). 

### CMD in patients without obstructive CAD, myocardial diseases and valvular diseases 

#### ANOCA and MVA

A large proportion of patients undergoing coronary angiography because of angina do not have obstructive epicardial coronary arteries (ANOCA). The mismatch between blood supply and myocardial oxygen demands leading to angina in ANOCA may be caused by CMD and/or epicardial coronary artery spasm (Vrints et al., 2024[115]).

MVA is the clinical manifestation of myocardial ischemia caused by CMD. MVA is characterized by effort-induced symptoms similar to those observed in patients with angina triggered by obstructive CAD. However, patients with MVA often also have angina at rest and a variable angina threshold, suggestive of dynamic coronary vasomotor changes (Kaski et al., 2018[46]). The COVADIS study group (Coronary Vasomotor Disorders) proposed the following diagnostic criteria for MVA: signs and symptoms of myocardial ischemia, reduced CFR (defined as the ratio of CBF during near maximal coronary vasodilatation to baseline CBF) or microvascular spasm, and documented myocardial ischemia, which is not triggered by obstructive CAD (Ong et al., 2018[78]). Historically, patients with MVA have been considered 'low risk' (Lamendola et al., 2010[49]), but recent invasive angiographic data demonstrate that this group includes a spectrum of patients, more frequently women, at higher risk of adverse cardiac events. The WISE study at 5.4-year follow-up demonstrated adverse events including cardiac death (53 % being sudden cardiac death), stroke, and new onset heart failure rather than myocardial infarction, in particular in women with reduced CFR assessed by adenosine (Pepine et al., 2010[83]). 

#### MINOCA

Myocardial infarction with non-obstructive coronary artery (MINOCA) is characterized by evidence of myocardial infarction (MI) in the absence of obstructive CAD. MINOCA accounts for up to 14 % of patients with acute MI. MINOCA patients are more likely to be younger and female compared to patients with obstructive CAD (Pasupathy et al., 2015[81]; Safdar et al., 2018[97]). CMD is considered to be a potential cause of MINOCA, even though not extensively studied as in stable chest pain patients (Tamis-Holland et al., 2019[106]). It has been demonstrated that MINOCA can be related to the luminal obliteration of coronary microcirculation due to thromboembolic mechanisms, resulting in CMD (Agewall et al., 2017[1]). Coronary thromboembolism starting directly from the left heart is typically caused by atrial fibrillation, valvular disease, intraventricular thrombi, and cardiac tumors. Another relevant possibility is debris from non-critical epicardial atherosclerotic plaque following spontaneous or iatrogenic rupture (Severino et al., 2020[100]). On the other hand, coronary thromboembolism can also be paradoxical in the presence of right-left shunt, such as in patent foramen ovale, atrial septal defect, and, rarely, arteriovenous fistula (Pilote and Karp, 2012[85]). Sometimes, microvascular spasm can be associated with acute presentation as MINOCA (Montone et al., 2018[65]).

Performing a diagnosis of the underlying cause of MINOCA is essential to guide a tailored therapy, with an ongoing trial evaluating whether a precision medicine approach compared to standard care could be associated with improved outcomes (Del Buono et al., 2021[20]; Montone et al., 2022[62]).

### CMD in Takotsubo syndrome

Takotsubo syndrome is an acute and usually reversible heart failure syndrome. By definition, patients with Takotsubo syndrome do not have obstructive CAD, yet they demonstrate abnormal myocardial perfusion (Elesber et al., 2006[24]), abnormal CFR measured by echocardiography (Rigo et al., 2009[91]), and abnormal PET imaging (Feola et al., 2006[30]), consistent with an inverse perfusion/ metabolism mismatch. However, these reported perfusion defects were consistently observed after cardiac dysfunction had developed. It is therefore unclear whether impaired coronary perfusion causes Takotsubo syndrome (TTS) in these cases, or whether perfusion defects occurred as a consequence of TTS and reduced diastolic function given the suction wave caused by myocardial relaxation is a main contributing factor to coronary perfusion (Davies et al., 2006[19]). In Takotsubo syndrome abnormal microvascular perfusion may be caused by reduced vasodilatation and increased vasoconstriction due to autonomic system imbalance, external microvascular compression due to myocardial edema and to increased intracardiac pressures (Redfors et al., 2014[89]). Therefore, although evidence from clinical studies and some preclinical studies suggests microvascular perfusion abnormalities are present during the acute phase of TTS, whether these are primary, or secondary to myocardial dysfunction, edema and inflammation, and how they contribute to the symptoms and pathophysiology remains to be determined (Omerovic et al., 2022[77]).

### CMD in patients with myocardial, infiltrative or valvular diseases

#### CMD in HFpEF

HFpEF is a heterogeneous syndrome characterized by classic symptoms and signs of heart failure (HF), a normal or near-normal ejection fraction (EF) and objective evidence of cardiac structural and/or functional abnormalities consistent with the presence of left ventricle (LV) diastolic dysfunction/raised LV filling pressures, including raised natriuretic peptides (McDonagh et al., 2021[56]). 

The PROMIS-HFpEF (Prevalence and correlates of coronary microvascular dysfunction in heart failure with preserved ejection fraction) study, which is the largest prospective study to date that was designed to determine the frequency of CMD in HpEF patients, investigated the prevalence of CMD and its association with endothelial dysfunction, myocardial dysfunction, and HF severity in patients with HFpEF. Nearly 75 % of patients in the study with a measurable CFR (n = 202) had evidence of CMD (CFR < 2.5). A low CFR was associated with several indices of HFpEF including higher N-terminal pro b-type natriuretic peptide, lower tricuspid annular plane systolic excursion, impaired right ventricular free wall strain, and evidence of systemic endothelial dysfunction (Shah et al., 2018[101]).

#### CMD in patients with hypertrophic cardiomyopathy 

Hypertrophic cardiomyopathy (HCM) is a primary myocardial disease, defined as an inappropriate ventricular hypertrophy that is disproportionate to the loading conditions. In HCM patients CMD may lead over time to recurrent ischemia and myocyte death, thus acting as a localizer of replacement fibrosis. Camici et al. first described an inadequate increase in MBF following the intravenous administration of dipyridamole in the majority of HCM patients studied with PET (Camici et al., 1991[10]). Similar to PET, a recent stress CMR study in HCM showed blunted myocardial blood flow (MBF) in response to stress. In addition, MBF was reduced to a greater degree in the subendocardial compared with the subepicardial layer, as in PET studies (Camici et al., 1991[10]), and also the degree of abnormal perfusion was related to the magnitude of wall thickness. Importantly, areas of myocardium in which fibrosis was present (as determined by late gadolinium enhancement) were most often associated with reduced MBF (Petersen et al., 2007[84]), supporting the hypothesis that abnormal MBF caused by microvascular dysfunction is responsible for myocardial ischemia-mediated myocyte death, and ultimately replacement fibrosis.

Several studies have suggested that CMD, as assessed by PET, increases the risk of sudden death and of a progressive and disabling clinical course with decline in left ventricular systolic function, impacting significantly the prognosis of HCM patients (Cecchi et al., 2003[12]; Nemes et al., 2009[72]; Olivotto et al., 2006[75]).

#### CMD in patients with infiltrative heart diseases

Anderson-Fabry disease (AFD) is a rare genetic lysosomal storage disorder caused by deficient activity of the enzyme α-galactosidase A, leading to progressive intracellular accumulation of neutral glycosphingolipids in different organs, including the heart. The cardiac deposition of globotriaosylceramide in myocytes, vascular endothelium, and smooth muscle cells leads to myocardial ischemia, myocardial wall thickening, and progressively to interstitial replacement fibrosis (Nakao et al., 1995[69]). CMD has a key role in AFD cardiomyopathy, accounting for the considerable prevalence of angina in the absence of obstructive CAD (Elliott et al., 2006[25]). Tomberli et al. showed that coronary microvascular function is markedly impaired in AFD patients irrespective of LV hypertrophy and sex and may also represent the first sign of the disease in patients with no other sign of cardiac involvement (Tomberli et al., 2013[109]).

Amyloidosis is a rare systemic disorder characterized by the extracellular deposition of misfolded protein in various organ systems including the heart. Anginal symptoms and signs of ischemia have been reported in some individuals with cardiac amyloidosis without obstructive epicardial CAD (Al Suwaidi et al., 1999[2]; Whitaker et al., 2004[116]). Autopsy studies have shown amyloid deposits around and between cardiac myocytes in the interstitium (Buja et al., 1970[6]), in perivascular regions (Modesto et al., 2007[59]), and in the media of intramyocardial coronary vessels (Hongo et al., 2000[44]). Thus, the pathophysiology of CMD is complex and involves structural (amyloid deposition in the vessel wall causing wall thickening and luminal stenosis), extravascular mechanisms (extrinsic compression of the microvasculature from perivascular and interstitial amyloid deposits and decreased diastolic perfusion), as well as functional (autonomic and endothelial dysfunction) mechanisms. Dorbala et al. demonstrated microvascular dysfunction via decreased myocardial blood flow and coronary flow reserve, using positron emission tomography, irrespective of the amyloid subtype (Dorbala et al., 2014[23]). 

#### CMD in patients with aortic stenosis

Half of the patients with a severe degree of aortic stenosis reports angina episodes, and this occurs despite the evidence of normal epicardial coronary arteries at angiography (Gould and Carabello, 2003[37]). Moreover, the occurrence of angina significantly increases the risk of sudden death compared to asymptomatic patients (Ross and Braunwald, 1968[95]). Marcus et al. showed a selective and marked decrease in coronary reserve in the coronary vessels that supplied the hypertrophied left ventricle in patients with severe aortic stenosis, suggesting that CMD is likely an important contributor to the pathogenesis of angina pectoris in these patients (Marcus et al., 1982[54]). CMD is reduced due to a combination of mechanisms including: (i) capillary rarefaction, (ii) reduced time of diastolic coronary filling, (iii) increased left ventricle diastolic pressure and intramyocardial pressure, (iv) low coronary myocardial pressure compared to intracavitary pressure.

Of interest, extravascular mechanisms rather than small vessel disease seem more responsible for the reduction of CFR in these patients, differently from patients with hypertrophic cardiomyopathy and those with left ventricular hypertrophy (LVH) secondary to systemic hypertension. Rajappan et al. showed that resting total left ventricular myocardial blood flow (MBF), measured through PET, increases proportionally with LV mass, despite reduced capillary density. It is likely that the increase in total LV blood flow is mainly sustained through metabolic vasodilatation in response to the increased oxygen demand of LVH. This is responsible for a partial exhaustion of the autoregulatory capacity of the coronary microcirculation and, therefore, contributes to reducing CFR. This study has also confirmed the experimental observation that sub-endocardial perfusion is severely reduced and that the severity of CMD was related to the aortic-valve area, imposed hemodynamic load, and diastolic perfusion time, rather than to left ventricular mass (Hittinger et al., 1995[43]; Rajappan et al., 2002[88]). Paolisso et al. confirmed these findings investigating coronary physiology using continuous thermodilution techniques. The increased resting flow, but unchanged hyperemic flow, resulted in abnormal CFR and microvascular resistance reserve (MRR) (Paolisso et al., 2022[80]).

## Therapeutic Strategies

To date, no large-scale randomized clinical trials have validated specific therapeutic strategies for CMD. Therefore, the management of patients with CMD should focus on addressing underlying risk factors and tailoring treatment based on their specific phenotypic presentation (Montone et al., 2024[63]).

Regarding risk factors, a strict control of traditional cardiovascular (CV) risk factors, such as hypertension, diabetes, smoking, dyslipidemia, is critical both in preventing as well as in improving CMD. Hypertension has been proved to be one of the most important causes of CMD and anti-hypertensive drugs have been demonstrated to be effective in some forms of MVA (Zdravkovic et al., 2023[119]). The main reason behind this finding is that hypertension has been closely linked to harmful adverse remodeling of coronary arterioles, thus keeping blood pressure well-controlled is essential for slowing down the progression of coronary microvascular disease and reduce both the frequency and severity of anginal symptoms (Engholm et al., 2016[27]; Kunadian et al., 2021[48]).

Cigarette smoking elevates resting myocardial blood flow, diminishes hyperemic flow and significantly lowers CFR. These vascular effects are more likely driven by tobacco-related microvascular dysfunction rather than perfusion deficits (Rooks et al., 2011[94]). 

Dyslipidemia is a well-known risk factor for the development of CMD. It has already been demonstrated that low density lipoprotein (LDL) cholesterol shows an inverse association with CFR, suggesting that LDL-related impairment of coronary microvascular function may critically contribute to the development and progression of coronary artery disease and its clinical complications (Kaufmann et al., 2000[47]). Its control through statins represents a primary goal to attain in order to reduce disease progression in CMD patients (Luo et al., 2019[53]).

As for diabetes, it has been proved to be a harmful risk factor not only for coronary atherosclerotic disease, but also for coronary microcirculation, since it induces microvascular damage. Hyperglycemia and insulin resistance can injure and impair repair processes of microvascular compartments due to the increase of metabolites associated with hyperglycemia in microvessels, the activation of inflammation cascade and the accumulation of toxic molecules, such as oxidants and advanced glycation end products (Salvatore et al., 2022[98]). Therefore, an effective prevention strategy involves aggressive management of diabetes, aimed at maintaining low blood glucose levels, controlling body mass index (BMI) with diet and exercise training, and reducing insulin resistance (Salvatore et al., 2022[98]).

More precisely, exercise training and weight loss have been demonstrated to improve prognosis in CMD patients, enhancing coronary endothelial function, endothelium-dependent vasodilation and coronary blood-flow reserve (Olsen et al., 2015[76]; Hambrecht et al., 2000[41]).

Also mental stress and acute and chronic psychological stress may elevate the risk of cardiovascular diseases (Montone et al., 2024[61]): in normal conditions, mental stress makes epicardial arteries and microvessels dilate, enhancing coronary blood flow to satisfy the higher oxygen demand. Instead, in patients with CMD, acute stress leads to a reduced ability of the resistance vessels to dilate, while the epicardial coronary arteries may constrict unexpectedly. As a result, the increased demand cannot be met by coronary flow, potentially resulting in myocardial ischemia (van der Meer and Maas, 2021[112]). Potential therapies for this condition relies on a pharmacological approach, constituted by stress reducing drugs such as antidepressants, or on a psychological approach, such as mindfulness exercises or meditation training (van der Meer and Maas, 2021[112]).

In addition to lifestyle changes, CMD patients have a considerable array of medications at their disposal (Table 2(Tab. 2); References in Table 2: Bairey Merz et al. 2016[4]; Houghton et al., 2000[45]; Mohri et al., 2003[60]; Morrow et al., 2024[68]; Neglia et al. 2011[71]; Russo et al., 2013[96]; Skalidis et al., 2011[102]; Suda et al., 2019[104]; Villano et al., 2013[114]). CorMicA trial has already demonstrated that a tailored therapy in patients with ischemia with no obstructive coronary disease - category in whom also CMD patients are included - brings to a longstanding improvement in angina symptoms and enhanced quality of life at one year from coronary angiography (Ford et al., 2020[32]).

Regarding the specific phenotype presentation, patients with CMD can be schematically divided into:

- Patients with microvascular spasm, where first line therapy is represented by non-dihydropyridines calcium antagonists (such as Verapamil or Diltiazem). Other useful drugs, which can be used in patients with refractory symptoms, are Nicorandil or Fasudil (Montone et al., 2024[67]).
- Patients with reduced CFR or increased IMR at functional coronary angiographic tests (“structural/functional group”), where first line therapies are represented by beta-blockers, calcium channel blockers, and angiotensin-converting-enzyme inhibitors (ACEi) or angiotensin receptor blockers (ARBs). Other effective drugs in this context are statins and some anti-anginal drugs such as Ranolazine, Ivabradine, and Fasudil (Vrints et al., 2024[115]). More precisely, in “functional” CMD, beta-blockers are the preferred treatment, whereas in “structural” CMD drugs like calcium channel blockers, Ivabradine, Nicorandil or Ranolazine should be preferred.

Beta-blockers are the first-line treatment for patients with CMD with effort-induced angina, especially considering that in those individuals an heightening of adrenergic activity is often present (Vrints et al., 2024[115]). Among beta-blockers, Nebivolol seems to be related to better outcomes if compared to the other drugs of the same class. The reason behind this evidence relies on the dual effect of this beta-blocker: a vasodilatory effect - given by its nitric oxide release mechanism - combined with a high b1-receptors selectivity, which makes it beneficial on exercise capacity. NIRVANA trial (NCT01665508), currently ongoing, is studying the effectiveness of this drug iin women with MVA.

Angiotensin-converting-enzyme inhibitors (ACEi) have been shown to act as a beneficial drug on microcirculation since they improve endothelial function and restore micro vessel blood flow, reducing angina symptoms (Montone et al., 2024[67]). The ESC 2024 guidelines for Chronic Coronary Syndromes classify ACEi as endothelial dysfunction and angina symptoms management drugs (Class IIa with a Level of Evidence B) (Vrints et al., 2024[115]).

A substudy of the Women's Ischemia Syndrome Evaluation (WISE) trial demonstrated a beneficial effect of Quinalapril on CFR in MVA women. This improvement was linked also to a clinical benefit, represented by a reduction in angina symptoms. The positive effect on the coronary microvasculature was mainly observed in women with lower baseline CFR values, indicating that the renin-angiotensin system may play a more significant role in those women with more severe microvascular damage (Pauly et al., 2011[82]).

Moreover, a study by Neglia et al. demonstrated that in hypertensive rats treated with Perindopril and Indapamide, those improvements which were detected in coronary flow after treatment were linked to the reversal of remodeling in intramural coronary arterioles and to an improvement of microvascular function (Neglia et al., 2011[71]).

Calcium channel blockers (CCBs) are the first line therapy for vasospastic angina (VSA) and for MVA due to microvascular spasm.

Their use is also relevant in “structural and functional” forms of microvascular angina (i.e., MVA with negative Ach test and impaired CFR, IMR or both), despite the conflicting evidence in the literature. A study by Sütsch et al. failed to demonstrate an increase in CFR in patients with MVA, in contrast with the commonly reported clinical benefits deriving from its use in CMD patients (Sütsch et al., 1995[105]).

Phosphodiesterase type 5 (PDE-5) inhibitors, commonly used drugs in erectile dysfunction and pulmonary hypertension, have been associated with a positive effect on CFR in MVA women. Another WISE substudy has shown that in women with CMD, PDE-5 inhibition is linked to a rapid improvement in CFR, with the effect being most pronounced in those with a CFR of 2.5 or lower. In women with a baseline CFR of 2.5 or lower, CFR increased from 2.1 ± 0.2 to 2.7 ± 0.6 (P = 0.006). In contrast, for women with a baseline CFR above 2.5, CFR did not show any change (from 3.1 ± 0.3 to 3.0 ± 0.6; P = 0.70). This result also demonstrated that the lower is the baseline CFR, the better is the outcome when CMD is treated with appropriate drugs (Denardo et al., 2011[21]). Statins also could improve CMD symptoms thanks to their anti-inflammatory and anti-atherosclerotic power. Of note, a study by Houghton et al., which investigated the effect of lipid lowering therapy with pravastatin on coronary resistance and endothelial function in six patients with angina and angiographically normal coronary arteries, demonstrated that - after 6 months of statin therapy - there was evidence of a significant improvement of coronary resistance and arterial endothelial function (Houghton et al., 2000[45]).

As for Ranolazine, a study by Bairey Merz et al. demonstrated that short-term late sodium current inhibition with Ranolazine in low coronary flow reserve (CFR <2.5) subjects improved myocardial perfusion reserve index and angina symptoms (as evaluated with Seattle Angina Questionnaire) (Bairey Merz et al., 2016[4]). Another study by Villano et al. found a statistically relevant efficacy of Ranolazine in symptomatic and stress test metrics, but no improvement in myocardial blood flow (Villano et al., 2013[114]). The same study showed some beneficial effects of Ivabradine in CMD patients, although its favorable effects were less relevant if compared with Ranolazine (Villano et al., 2013[114]).

On the other hand, a study by Skalidis et al. has shown an Ivabradine related improvement of CFR along with a heart rate lowering effect. Of note, this study demonstrated that the increase of CFR was independent from heart rate reduction since its values were constant at a coronary functional test carried out one week after treatment start, both at Ivabradine induced bradycardia and at a paced heart frequency identical to that before treatment (Skalidis et al., 2011[102]). In this context, Ranolazine and Ivabradine may serve a therapeutic alternative for MVA patients who are experiencing insufficient symptom control when used alongside standard anti-ischemic treatment (Villano et al., 2013[114]).

As for long acting nitrates, there is no evidence demonstrating a clinical benefit in MVA patients. Furthermore, they are considered potentially harmful in this category of patients since they could provide coronary steal mechanism in the microcirculation or reduce nitrates responsiveness (Russo et al., 2013[96]).

Similarly, short acting nitrates, although useful in cases of angina exacerbations in patients with a reduced vasodilatory capacity, are only partially beneficial (Crea et al., 2014[14]).

Xanthine-derivative, such as caffeine, aminophylline, paraxanthine, pentoxifylline, theobromine, and theophylline, could have some beneficial effects on CMD patients. A work by Crea et al. already demonstrated Aminophylline effectiveness on exercise-induced chest pain, despite being ineffective on ST segment depression (Crea et al., 1990[18]). The same result was reached by a randomized trial by Elliot et al. which showed a positive effect on the threshold for exercise-induced chest pain despite differing and various impacts on ST segment changes (Elliott et al., 1997[26]). The analgesic effect of xanthine-derived compounds could be driven both by antagonization of adenosine P-receptors - normally associated with response to nociceptive stimuli - and by inhibition of adenosine A-2 receptors, which cause arteriolar dilation and whose blockage may have a beneficial effect in redistributing coronary blood flow toward ischemic regions (Crea et al., 1994[15]).

Moreover, Fasudil, a potent Rho-kinase inhibitor, could represent a novel and effective therapy in MVA patients. Indeed, a study by Mohri et al. demonstrated that this drug was effective in those patients with microvascular angina in whom coronary microvascular spasm is causatively involved (Mohri et al., 2003[60]). A more recent study by Suda et al. demonstrated that in CMD patients, the use of Fasudil importantly ameliorated IMR of the treated group, if compared with the other 3 control groups of the study (Suda et al., 2019[104]).

Finally, also Endothelin-1, an endogenous vasoconstrictor, has been involved in coronary microvascular dysfunction. It has already been demonstrated that this molecule is connected with endothelial dysfunction and inflammation (Ford et al., 2020[31]); moreover, levels of circulating Endothelin-1 have been demonstrated to inversely correlate with coronary flow reactions in CMD patients (Halcox et al., 2007[40]). Nevertheless, the randomized, placebo-controlled Precision Medicine With Zibotentan in Microvascular Angina (PRIZE) trial, which aimed to demonstrate a beneficial effect provided by Zibotentan, an oral Endothelin-A receptor selective antagonist, showed that short-term treatment with a daily low dose of Zibotentan was ineffective, and target-related adverse effects were frequently observed (Morrow et al., 2024[68]).

## Multi-omics Analyses

Multi-omics analyses are a novel approach towards the study of human diseases, integrating several datasets represented by genomics, single-cell transcriptomics, proteomics and metabolomics (Chen et al., 2023[13]). In recent years, both clinical and pre-clinical studies have used multi-omics analyses to evaluate CMD. Smilowits et al. investigated whole-blood transcriptome in 28 woman with INOCA showing that individuals with CMD defined by a low CFR at thermodilution had a proinflammatory whole blood transcriptional signature (Smilowitz et al., 2024[103]). Another study supporting the hypothesis that inflammation may underlie CMD was conducted by Prescott et al., who quantified 184 unique cardiovascular proteins in 1471 women with ANOCA and CMD evaluated by CFVR by transthoracic echo Doppler. CMD was associated with pathways involving inflammation (interleukin 6), blood pressure and ventricular remodeling (BNP/NT-proBNP) (Prescott et al., 2023[86]). In preclinical models too, multi-omics analysis of the cardiac cellulome have been offering new insights on the pathophysiology and possible therapeutic CMD targets. For instance, Lu et al. showed changes in myocardial metabolome and acetylated proteome in CMD rats (Lu et al., 2024[52]), while Dona et al. demonstrated critical involvement of smooth muscle cell-mineralocorticoid receptor in obesity associated coronary and cardiac dysfunction in obese female mice (Dona et al., 2023[22]).

## Future Perspectives

CMD is a complex, multifaceted condition that plays a pivotal role in myocardial ischemia, heart failure, and adverse cardiovascular outcomes. Despite increasing recognition of its clinical significance, CMD remains underdiagnosed and undertreated due to the limitations of conventional diagnostic strategies and the lack of standardized therapeutic approaches. While advances in invasive and non-invasive techniques, such as intracoronary physiological testing and myocardial perfusion imaging, have enhanced diagnostic precision, their routine clinical adoption remains limited. Future research should prioritize large-scale randomized clinical trials to develop evidence-based treatment strategies tailored to CMD phenotypes. Integrating multi-omics analyses, artificial intelligence, and patient-derived models within a precision medicine approach may allow for personalized therapeutic interventions, ultimately improving outcomes for patients with CMD.

## Declaration

### Conflict of interest

None.

### Funding

None.

### Acknowledgments

**Figure 4:**
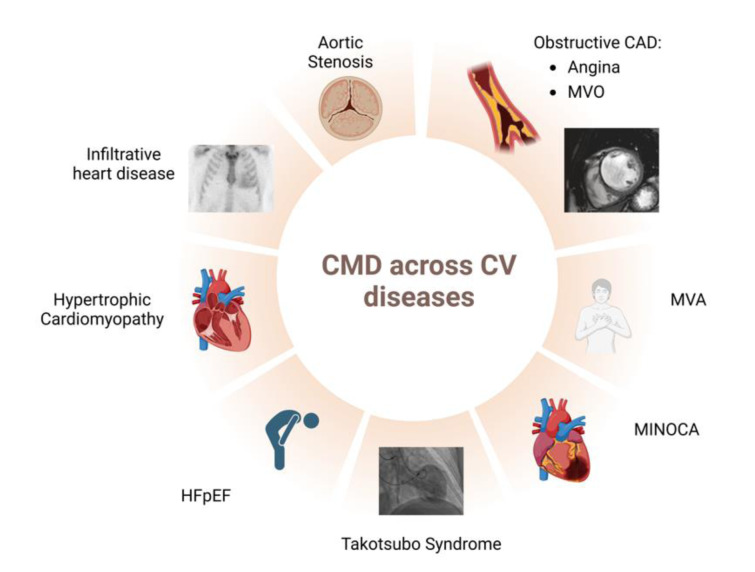
The role of CMD across cardiovascular diseases Abbreviations. CAD: coronary artery disease; HFpEF: Heart failure with preserved ejection fraction; MINOCA: myocardial infarction and non-obstructive coronary arteries; MVA: microvascular angina; MVO: microvascular obstruction.

Figures 1(Fig. 1), 2(Fig. 2), 3(Fig. 3) and 4(Fig. 4) were created with Biorender.com.

### Using Artificial Intelligence (AI)

We have not used artificial intelligence (AI)-assisted technologies in the production of the submitted work.

## Figures and Tables

**Table 1:**
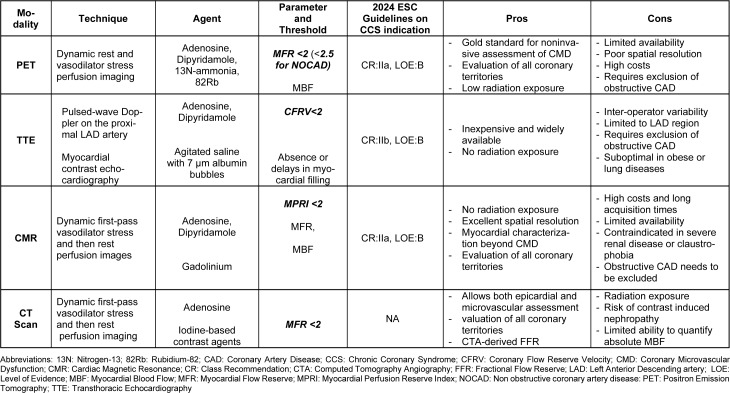
Non-invasive techniques for the diagnosis of CMD

**Table 2:**
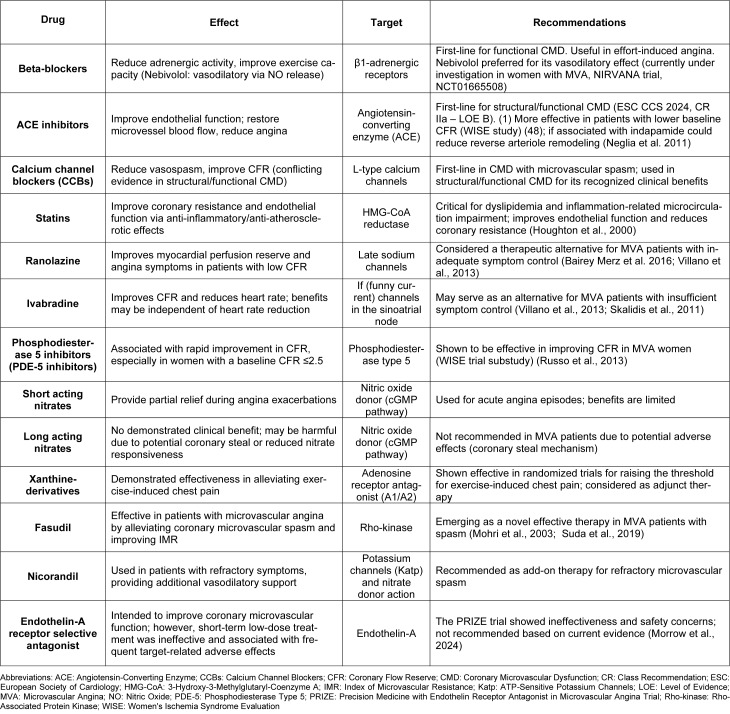
Current therapeutic strategies for microvascular dysfunction
